# Association of Acupuncture and Auricular Acupressure With the Improvement of Sleep Disturbances in Cancer Survivors: A Systematic Review and Meta-Analysis

**DOI:** 10.3389/fonc.2022.856093

**Published:** 2022-05-18

**Authors:** Qingyun Wan, Shuting Luo, Xiaoqiu Wang, Qianmo Tian, Hanqing Xi, Shiyu Zheng, Qinqin Fang, Hao Chen, Wenzhong Wu, Rui Pan

**Affiliations:** ^1^ Department of Acupuncture and Rehabilitation, Jiangsu Province Hospital of Chinese Medicine, Affiliated Hospital of Nanjing University of Chinese Medicine, Nanjing, China; ^2^ Department of Acupuncture and Rehabilitation, Nanjing Traditional Chinese Medicine Hospital, Affiliated Hospital of Nanjing University of Chinese Medicine, Nanjing, China; ^3^ The Second Clinical Medical College, Nanjing University of Chinese Medicine, Nanjing, China; ^4^ Department of Oncology, Jiangsu Cancer Hospital, Affiliated Cancer Hospital of Nanjing Medical University, Nanjing, China

**Keywords:** sleep disturbances in cancer survivors, acupuncture, auricular acupressure, systematic review, meta-analysis

## Abstract

**Background:**

Studies on the efficacy of acupuncture and auricular acupressure on sleep disturbances in cancer patients have been growing, but there is no specific and comprehensive systematic review and meta-analysis. This review aims to evaluate the efficacy and safety of acupuncture and auricular acupressure on sleep disturbances in cancer survivors based on existing randomized clinical trials (RCTs).

**Methods:**

Four English-language and four Chinese-language biomedical databases were searched for RCTs published from database inception to July 30, 2021. RCTs comparing acupuncture and auricular acupressure with sham control, drug therapy, behavior therapy, or usual care for managing cancer were included. The quality of RCTs was appraised with the Cochrane Collaboration risk of bias (ROB) tool. Mean differences (MDs) and 95% confidence intervals (CIs) were calculated for the effect sizes.

**Results:**

Thirteen RCTs with 961 patients were included. The risk of performance bias or reporting bias for most of the included trials was high or unclear. Evidence was not found for short-term effects on sleep scales compared to sham control (MD, 1.98; 95% CI, 0.33–3.64; *p* = 0.02; *I^2^* = 36%), wait list control (MD, 0.40; 95% CI, −0.87–1.68; *p* = 0.54; *I^2^* = 49%), drug therapy (MD, 1.18; 95% CI, −3.09–5.46; *p* = 0.59; *I^2^* = 98%). For long-term effect, two sham-controlled RCTs showed no significance of acupuncture on insomnia scale scores (MD, 1.71; 95% CI, −2.38–5.81; *p* = 0.41; *I^2^* = 89%). Subgroup analyses suggested no evidence that auricular acupressure (MD, 3.14; 95% CI=1.52, 4.76; *p* = 0.0001; I^2^ = 0%) or acupuncture (MD, 0.54; 95% CI=−1.27, 2.34; *p* = 0.56; I^2^ = 0%) was associated with the reduction in insomnia scale scores.

**Conclusions:**

This systematic review and meta-analysis found no evidence about acupuncture or auricular acupressure in the improvement of sleep disturbances in cancer survivors in terms of short- or long-term effect. Adverse events were minor. The finding was inconsistent with previous research and suggested that more well-designed and large-scale randomized controlled trials are needed to identify the efficacy of acupuncture and auricular acupressure for sleep disturbances in cancer survivors.

**Systematic Review Registration:**

https://www.crd.york.ac.uk/prospero/, CRD42020171612.

## 1 Introduction

Sleep disorder is one of the most prevalent symptoms in patients with cancer (1, 2). It is reported that the prevalence of insomnia in cancer ranges from 30% to 50%, nearly three times higher than that in the general population (3). Although symptoms may slightly improve with time, most of the patients still suffer from insomnia years after cancer treatment (4, 5). Side effects of cancer treatment like depression and pain led to insomnia (6); meanwhile, insomnia would affect cancer treatment effects and result in a decreased quality of life with daytime fatigue, poor cognition, emotion disorder, and other health problems (7, 8). Unfortunately, sleep disturbances in cancer surviovrs are often underrecognized and poorly managed (9).

Pharmacological therapy is the typical options for chronic insomnia, but many cancer survivors refuse to take these drugs taking into account the side effects and drug-to-drug interactions in case of polypharmacy and dependency (10, 11). Instead, cognitive-behavioral therapy (CBT) is the first-line treatment for chronic insomnia yet can be difficult to access, which may require trained therapists to address survivorship-specific barriers to sleep (12). Thus, it is urgent to find a widely used, relatively safe, and well-accepted intervention. Both acupuncture and auricular acupressure (AA), as complementary and alternative treatment, might be promising treatment options, which appear to be effective in improving cancer-related insomnia (13, 14). Acupuncture has been defined as the insertion of fine needles into specific acupuncture points (acupoints) in the human body to relieve certain symptoms, and AA is a non-invasive therapy of treating physical and psychosomatic diseases by stimulating specific points of ears with something (15). Studies on acupuncture and AA for cancer-related insomnia have been growing. There were two systematic reviews discussing the association of acupuncture with cancer-related insomnia. One was published in 2013 and only included three studies (16), and the other one was published in 2016 and included six trials. However, two trials (17, 18) included in the review were conducted by one team, and data were repeated (19). Meanwhile, there were four new trials conducted in the years since 2016 (20–23). Thus, we pooled data from previous RCTs and conducted a systematic review and meta-analysis to investigate the efficacy and safety of acupuncture and AA on sleep in patients with cancer.

## 2 Methods

This systematic review and meta-analysis was registered in PROSPERO (No. CRD42020171612) and followed a pre-specified analysis plan.

### 2.1.Eligibility Criteria

Study eligibility was assessed according to five aspects of PICOS.

*Participants.* Patients who were diagnosed with cancer with or without sleep disturbances were included, but sleep quality was estimated pre- and post-treatment with records of sleep.

*Interventions*. Interventions included acupuncture and/or AA regardless of specific needling techniques such as manual acupuncture, electroacupuncture, or a combination of acupuncture or AA with other therapies for sleep disturbances.

*Comparisons*. Sham acupuncture or acupressure, usual care for managing cancer symptoms, drug therapy, or cognitive behavioral therapy were compared. Studies comparing different acupuncture techniques (i.e., warming needle acupuncture vs. manual acupuncture) or different acupoint combinations (i.e., scalp acupuncture vs. body acupuncture) were excluded.

*Outcomes.* Outcome measures were as follows: first, scales or parameters for sleep evaluation, such as Pittsburgh Sleep Quality Index (PSQI), Insomnia Severity Index (ISI), and other scales including items reflecting the severity of insomnia such as cancer quality of life questionnaire-core 30 (QLQ-C30), M.D. Anderson symptom inventory (MDASI), quality of life (QOL); second, logbook or sleep diary with records of sleep assessment; and third, adverse events. Studies that did not test the efficacy of acupuncture or AA on sleep disorder in cancer patients were excluded.

*Studies*. Studies that were included were randomized controlled trials.

### 2.2 Search Strategy

Four English databases (PubMed, Embase, Cochrane Library, and Web of Science) and four Chinese databases (China National Knowledge Infrastructure, Wanfang Database, VIP Database for Chinese Technical Periodicals, and Chinese Biomedical Literature Database) were searched for RCTs published from the database inception to July 30, 2021. The search terms included (acupuncture OR acupuncture therapy OR auricular acupressure) AND (cancer OR tumor OR neoplasms OR carcinoma OR malignancy) AND (sleep initiating and maintaining disorders, insomnia OR sleep disturbance OR sleep disorder OR wakefulness OR waking) AND (randomized controlled trial OR controlled clinical trial) in Chinese and English. The details of the search strategy are shown in **Table 1**.

**Table 1:** Search strategy used for the PubMed database.

Number	Search items
#1	Neoplasms [Mesh] OR Neoplasia [Title/Abstract] OR Tumor [Title/Abstract] OR Cancer [Title/Abstract] OR Malignancy [Title/Abstract] OR Malignant Neoplasm [Title/Abstract] OR Benign Neoplasm [Title/Abstract]
#2	Sleep Initiating and Maintaining Disorders [Mesh] OR Early Awakening [Title/Abstract] OR Nonorganic Insomnia [Title/Abstract] OR Primary Insomnia [Title/Abstract] OR Transient Insomnia [Title/Abstract] OR Rebound Insomnia [Title/Abstract] OR Secondary Insomnia [Title/Abstract] OR Sleep Initiation Dysfunction [Title/Abstract] OR Sleeplessness [Title/Abstract] OR Insomnia Disorder [Title/Abstract] OR Insomnia [Title/Abstract] OR Chronic Insomnia [Title/Abstract] OR Psychophysiological Insomnia [Title/Abstract]
#3	#1 AND #2
#4	Acupuncture [Mesh] OR Acupuncture therapy [Mesh] OR Acupuncture Treatment [Title/Abstract] OR Acupuncture, Ear [Mesh] OR Auricular Acupuncture [Title/Abstract] OR Acupuncture points [Mesh] OR Acupoints [Title/Abstract]
#5	#3 AND #4
#6	Randomized Controlled Trials as topic [Mesh] OR Clinical Trials, Randomized [Title/Abstract] OR Controlled Clinical Trials, Randomized[Title/Abstract] OR Randomized Controlled Trial [Publication Type] OR Intention to Treat Analysis [Mesh] OR Controlled Clinical Trials as Topic [Mesh]
#7	#5 AND #6

### 2.3 Study Selection

Two of the authors (HX and QF) independently identified articles by scanning titles and abstracts and full text as needed. A third reviewer (QT) would be involved to resolve disagreements between two reviewers by consensus. Articles meeting criteria would be included in the systematic review and, if applicable, included in the meta-analysis.

### 2.4 Data Extraction

All data extraction was independently performed by two reviewers (HX and QF) using a predetermined protocol according to Preferred Reporting Items for Systematic Reviews and Meta-Analyses (PRISMA) reporting guidelines (24). These variables included details of trials (year of publication, country, study design, and number of participants), participants (age, sex, cancer type, cancer treatment, and treatment status), intervention (acupoints, frequency, and course), comparisons, and outcomes (sleep measures and adverse event).

For the insomnia scores, the results measured by other scales were converted to the corresponding grade in the 21-point PSQI (higher points indicating more severe insomnia). For example, the result of a 3-score on the 10-score version of the MDASI sleep item was recorded as 6.3 points for data synthesis. The results of the PSQI, the converted QLQ-C30, MDASI, and QOL were used in the meta-analysis. Data in other forms (i.e., mean [95% CI], median, and interquartile range) were converted to means (SDs) according to the Cochrane Handbook for Systematic Reviews of Interventions (25). Corresponding authors would be contacted if data (i.e., SDs) acquisition failed in the original article.

### 2.5 Quality Assessment

The quality of RCTs included was appraised with the Cochrane Collaboration risk of bias tool (25). Each RCT was assigned a low, high, or unclear risk of bias (ROB) for six specific domains (sequence generation, allocation concealment, blinding of participants and outcome assessment, incomplete outcome data, selective outcome reporting, and other potential threats), with information originating from the published articles.

### 2.6 Data Synthesis and Analysis

Meta-analysis of RCTs with data was performed by calculating the effect size and 95% CI using the random-effects model. When the number of studies was less than five or studies were substantially heterogeneous, the random-effects model was according to Cochrane Handbook for Systematic Reviews of Interventions (25). Statistical heterogeneity across trials was assessed and quantified by the *I^2^* statistic using forest plots. *I^2^* value >50% was substantially heterogeneous. Statistical analysis was performed with RevMan, version 5.3. Two-sided *p* < 0.05 was considered statistically significant.

Studies were grouped according to the comparators including sham therapy, drug therapy, behavior therapy, and usual care for managing cancer insomnia. For studies with over one control group, such as real acupuncture vs. sham acupuncture vs. wait-list control, the results were split into corresponding comparison groups.

We conducted subgroup sensitivity analysis to explore potential sources of heterogeneity. Planned subgroup analysis was the type of intervention including manual acupuncture and AA.

## 3 Results

**Figure 1:**
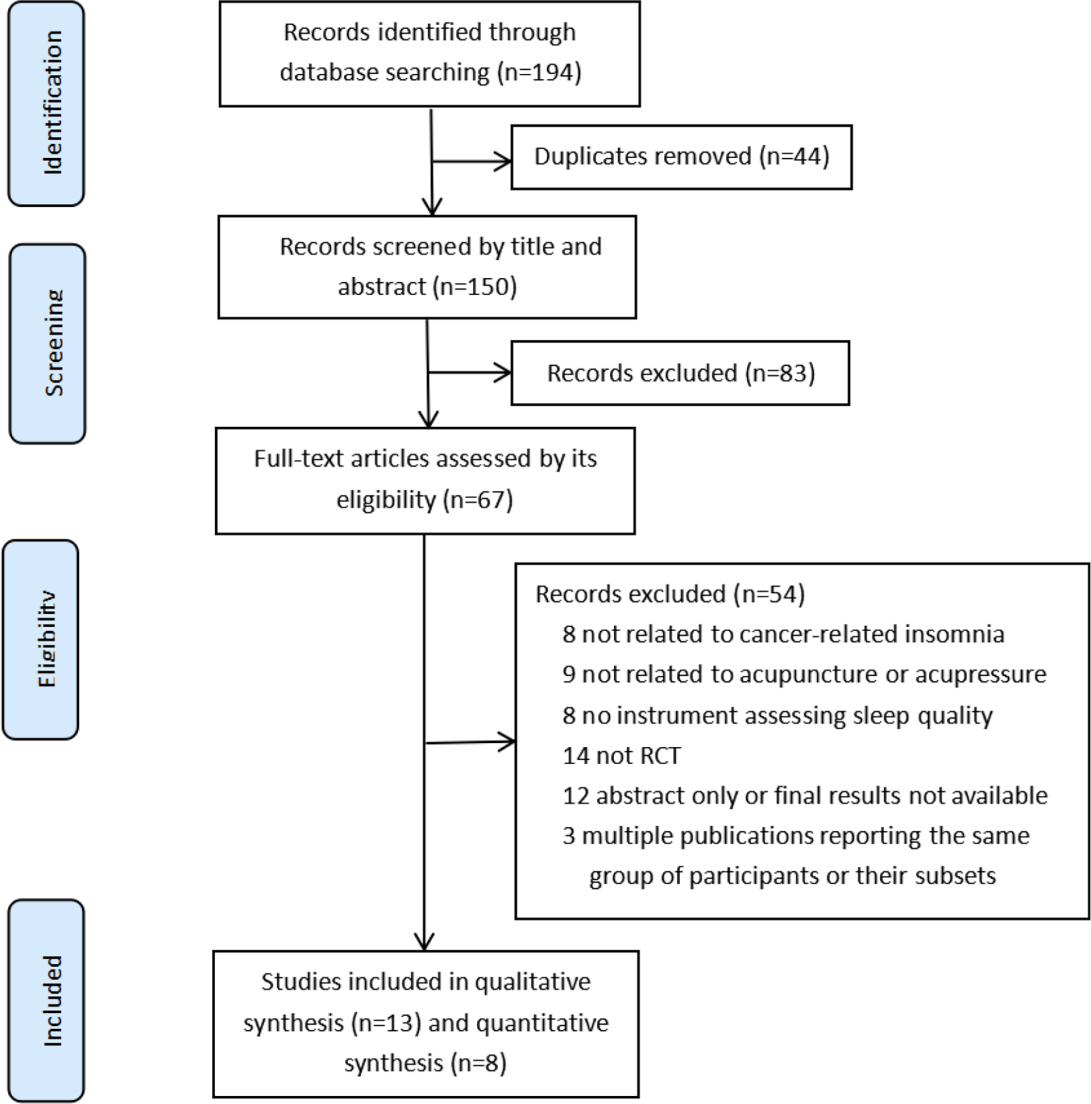
PRISMA flow diagram.

A total of 194 studies were identified by database search. Of those, 44 duplicates and 83 records were filtered and discarded through titles and abstracts; 54 records were excluded for other reasons (**Figure 1**). At last, 13 RCTs (20–23, 26–34) met our inclusion criteria and were included in this review, only 8 RCTs (20, 27–31, 33, 34) with sufficient data were included in the meta-analysis.

### 3.1 Study Characteristics

Characteristics of trials, patients, interventions, comparators, and outcomes are shown in **Tables 2**, **3**.

**Table 2:** Characteristics of studies and patients.

Source	Country	Study design	No. of patients (E/C)	FemalePercent (E/C)	Mean age (SD)(E/C)	Cancer type	Cancer treatment	Treatment status	Inclusion about sleep status
**Acupuncture or acupressure vs. sham control**									
Bokmand et al., 2013 (26)	Denmark	RCT three-arms	31/29	1/1	60/62	Breast cancer	Surgery, chemotherapy	Off cancer treatments	Self-estimated disturbed night sleep
Lu et al., 2012 (27)	America	Pilot RCT	11/10	1/1	50.8 ± 10.6 /50 ± 9.9	Ovarian cancer	Chemotherapy	Undergoing standard chemotherapy	NR
Mao et al., 2014 (28)	America	RCT three-arms	22/22	1/1	57.5 ± 10.1 /60.9 ± 6.5	Breast cancer	Aromatase inhibitor	Receiving an aromatase inhibitor	NR
Yeh et al., 2016 (29)	America	RCT	16/15	1/1	58.32 ± 10.93	Breast cancer	Chemotherapy, radiotherapy	Having completed the first cycle of cancer treatment	Reporting on a 0–10-point numeric rating scale
Yoon et al., 2019 (20)	South Korea	RCT	20/21	1/1	45.05± 8.33 /44.57 ± 8.34	Breast cancer	Surgery, chemotherapy	Undergoing anthracycline chemotherapy with a 3-week cycle after breast cancer surgery	ISI score ≥ 8
**Acupuncture or acupressure vs. wait list control**									
Bokmand et al., 2013 (26)	Denmark	RCT three-arms	31/24	1/1	60/62	Breast cancer	Surgery, chemotherapy	Off cancer treatment	Self-estimated disturbed night sleep
Mao et al., 2014 (28)	America	RCT three-arms	22/23	1/1	57.5 ± 10.1 /60.6 ± 8.2	Breast cancer	Aromatase inhibitor	Receiving an aromatase inhibitor	NR
Meng et al., 2010 (30)	China	RCT	44/41	43%/46%	54.3/53.1	Colon cancer	Intraperitoneal surgery	Postoperative	NR
**Acupuncture or acupressure vs. drug**									
Feng et al., 2011 (31)	China	RCT	40/40	65%/67.5%	63.80 ± 5.47 /63.60 ± 4.26	Malignant tumor	NR	NR	PSQI score ≥ 8
Frisk et al., 2012 (32)	Sweden	RCT	26/18	1/1	54.1/53.4	Breast cancer	NR	Having completed treatment for breast cancer *in situ*	NR
Garland et al., 2017 (33)	America	RCT	30/28	1/1	52.9 ± 8.6 /50.4± 8.4	Breast cancer	NR	Free of cancer treatment	NR
Peng et al., 2016 (34)	China	RCT	93/97	34.6%/34.6%	59.36 ± 12.54 /60.9 ± 11.11	NR	NR	NR	Meeting the criteria for insomnia disorder defined by TCMDEC
**Acupuncture or acupressure vs. CBT**									
Garland et al., 2019 (21)	America	RCT	80/80	53.8%/60%	62.3 ± 11.4 /60.7 ± 12.0	NR	Surgery, chemotherapy, radiotherapy	Have completed active treatment at least 1 month prior to study initiation	ISI score > 7, and meeting the criteria for insomnia disorder defined by DSM-5
**Acupuncture or acupressure plus drug vs. drug**									
Deng et al., 2019 (22)	China	RCT	30/30	63.3%/66.7%	53 ± 9/49 ± 11	Malignant tumor	Chemotherapy	Receiving chemotherapy	NR
**Acupuncture or acupressure plus sleep hygiene practices vs. sleep hygiene practices**									
Kuo et al., 2018 (23)	Australia	Pilot RCT	20/20	1/1	51.56 ± 13.23 /54.73 ± 14.56	Ovarian cancer	Chemotherapy	Receiving chemotherapy	PSQI score > 5, and no history of sleep disorders

E/C, experimental group/control group; RCT, randomized controlled trial; PSQI, Pittsburgh sleep quality index; ISI, insomnia severity index; CBT, cognitive behavior therapy; NR, not reported.

**Table 3:** Characteristics of interventions, comparators, and outcomes.

Source	Intervention	Acupoints	Frequency	Duration of intervention	Comparator	Follow up	Outcome measures	Outcomes	Adverse event
**Acupuncture or acupressure vs. sham control**									
Bokmand et al., 2013 (26)	AT	HC6, KI3, SP6, and LR3	Once a week	5 weeks	SA, choosing four pre-determined bilateral non-acupuncture points outside the meridians, and inserted superficially in the skin. The frequency and duration were the same as the intervention.	6, 12 weeks after the last treatment	Disturbed night sleep, rated “yes” or “no” at the same time points.	Significant short- and long-term effects.	No side effects
Lu et a.l, 2012 (27)	EA	GV20, SP10, ST36, SP6, KI3, LR3	2–3 times per week	10 sessions of acupuncture treatment	SA, using 5 non-acupuncture points off the meridians, minimally inserted, no hand manipulation and De Qi were allowed. The frequency and duration were the same as the intervention.	None	QLQ-C30	No significant effects.	No adverse effects
Mao et al., 2014 (28)	EA	At least four local points around the joint with the most pain and at least four points to address constitutional symptoms	Twice a week for 2 weeks, then weekly for six more weeks	8 weeks	SA, performed using Streitberger non-penetrating needles at non-acupuncture, non-trigger points at least 5 cm from the joint where pain was perceived to be maximal. The frequency and duration were the same as the intervention.	4 weeks post-treatment	PSQI	No group comparison.	NR
Yeh et al., 2016 (29)	AA	Shenmen, sympathetic, occiput, subcortex nervous, neurasthenia points, anxious, corresponding points related to location of body pain (varied for each participant)	Once a week	4 weeks	Sham AA, the stomach, mouth, duodenum, and eye acupoints unrelated to the symptom cluster of interest. The frequency and duration were the same as the intervention.	4 weeks after the last treatment	MDASI	No significant effects.	Participants reported minimal, adverse local effects from the AA treatment.
Yoon et al., 2019 (20)	AA	Shenmen, heart, anterior lobe, occiput	6 times a week	6 weeks	Sham AA, 4 points of helix located in the auricle. The frequency and duration were the same as intervention.	None	PSQI	Significant short-term effects.	No side effects
**Acupuncture or acupressure vs. WLC**									
Bokmand et al., 2013 (26)	AT	HC6, KI3, SP6, and LR3	Once a week	5 weeks	No treatment	6, 12 weeks after the last treatment	Disturbed night sleep, rated “yes” or “no” at the same time points.	Significant short-term and long-term effects.	No side effects
Mao et al., 2014 (28)	EA	At least four local points around the joint with the most pain and at least 4 points to address constitutional symptoms	Twice a week for 2 weeks, then weekly for six more weeks	8 weeks	No treatment	4 weeks post-treatment	PSQI	No significant effects.	NR
Meng et al., 2010 (30)	EA	SJ6, GB34, ST36, ST37	Once a day, starting on postoperative day 1	for six consecutive days or until the first bowel movement	Usual care	None	QOL	No significant effects.	No adverse effects
**Acupuncture or acupressure vs. drug**									
Feng et al., 2011 (31)	AT	ST40,SP9,SP10,SP6,EX-HN3,DU20,EX-HN1,PC6,TF4	One time per day	30 days	Fluoxetine hydrochloride capsule, 20 mg/day for 30 days	None	PSQI	Significant short-term effects.	NR
Frisk et al., 2012 (32)	EA	BL15,BL23,BL32,GV20,HE7,PC6,LR3, SP6, SP9	2 times weekly for first 2 weeks, then 1 time weekly for 10 weeks	12 weeks	Hormone therapy, treated with sequential or continuous combined estrogen/progestagen therapy for 24 months.	A total of 24 months, being assessed every 3 months.	Times woken up/night and hours slept; WHQ sleep score	No group comparison.	No adverse event
Garland et al., 2017 (33)	EA	Standard points depending on subjects’ preferred positions and up to four acupuncture points chosen on the basis of subjects’ other presenting symptoms	Twice a week for 2 weeks, then weekly for six more weeks.	8 weeks	Gabapentin, a 6-day titration phase when participants took one pill(300 mg) at bedtime for 3 days, then twice daily for 3 days, and then three times daily for the remaining 50 days (a total of 8 weeks).	None	PSQI	Significant short-term effects.	NR
Peng et al., 2016 (34)	AT and moxibustion	DU20,DU24,EX-HN3,HT7,ST36,SP6,RN4,RN14	One time per day	7 days	Estazolam, 1 mg/day for 7 days.	7 days post-treatment	PSQI, sleep effective rate.	Significant short- and long-term effects, but drug was more effective for short-term effects.	NR
**Acupuncture or acupressure vs. CBT**									
Garland et al., 2019 (21)	AT	Standardized points commonly used to address sleep problems with additional points to treat comorbid symptoms like pain and anxiety if needed	Twice weekly for 2 weeks, then weekly for six more weeks	8 weeks	CBTI, five weekly sessions followed by two bi-weekly sessions, for seven total sessions over 8 weeks.	3 months after treatment	PSQI, ISI	Significant short- and long-term effects, but CBTI was more effective.	All adverse events were mild to moderate.
**Acupuncture or acupressure plus drug vs. drug**									
Deng et al, 2019 (22)	AT and Sertraline Hydrochloride Tablets	LI4,LR3,PC6,HT7,ST36,SP6,GV20,GV29	Acupuncture for twice a week based on control treatment	4 weeks	Sertraline hydrochloride tablets, 25 mg/day for the first 5 days, and 50 mg/day for next days, for 4 consecutive weeks.	None	QLQ-C30	Significant short-term effects.	No obvious adverse events
**Acupuncture or acupressure plus sleep hygiene practices vs. sleep hygiene practices**									
Kuo et al., 2018 (23)	AA and sleep hygiene practices	Shenmen, Xin, Pizhixia, and Neifenmi	3 times per day	6 weeks	Sleep hygiene practices.	None	PSQI	Significant short-term effects.	NR

AT, acupuncture; EA, electroacupuncture; SA, sham acupuncture; WLC, wait-list control; WHQ, Women’s Health Questionnaire; QLQ-C30, Cancer-Quality of Life Questionnaire-Core 30; MDASI, M.D. Anderson Symptom Inventory; QOL, quality of life.

Studies were conducted in six countries: five studies (21, 27–29, 33) in America, four studies (22, 30, 31, 34) in China, and one each in South Korea (20), Denmark (26), Sweden (32), and Australia (23). Among the included studies, only two of them (15%) were three-arms RCTs (26, 28) with two comparators of sham control and wait-list control.

The sample size of patients ranged from 21 to 190, and we included 961 patients with 463 in the experimental group and 498 in the comparator group. The mean age of participants varied from 44 to 63 years old. The types of cancer involved breast cancer (46%) (20, 26, 28, 29, 32, 33), ovarian cancer (15%) (23, 27), malignant tumor (15%) (22, 31), colon cancer (15%) (30), and unspecific cancer (15%) (21, 34). Five of the retrieved studies (52%) included patients undergoing cancer treatments (chemotherapy, radiotherapy, surgery, and cancer-related therapies) at the time of the study (20, 22, 23, 27, 28), while four studies were conducted after treatment (26, 28, 30, 33); three other studies (30%) investigated only patients having completed at least one cycle of cancer treatment (21, 29, 32). Four studies (30%) gave clear scaled inclusion criteria for sleep disturbances with the lowest scores of PSQI or ISI ranging from 5 to 10 (20, 21, 23, 31). There were six trials (46%) that assessed the sleep quality of cancer patients before and after treatment without inclusion criteria for insomnia (22, 27, 28, 30, 32, 33).

Interventions among the included studies were shown as single forms (acupuncture, electro-acupuncture, auricular acupressure) or combinations (acupuncture and sertraline hydrochloride tablets, auricular acupressure, and sleep hygiene practices), only with clear disparities in acupoints, frequency, and duration. All studies were divided into six groups according to the intervention and comparator: five studies in the sham-controlled group (20, 26–29), three studies in the wait-list control group (26, 28, 30), four studies in the comparator group of drug (31–34), and one each in the group of acupuncture compared to CBT (21), acupuncture plus drug compared to the drug (22), and auricular acupressure plus sleep hygiene practices compared to sleep hygiene practices (23).

Outcome measures were the use of logbook, where disturbed night sleep or times woken at night were recorded in one RCT (26, 32), and sleep scales (i.e., PSQI, ISI, and QLQ-C30). PSQI was applied in seven trials (20, 21, 23, 28, 31, 33, 34). QLQ-C30 was used in two RCTs (22, 27); MADSI (29) and QOL (30) were used in one study, respectively.

### 3.2 Risk of Bias in Individual Studies

**Figure 2:**
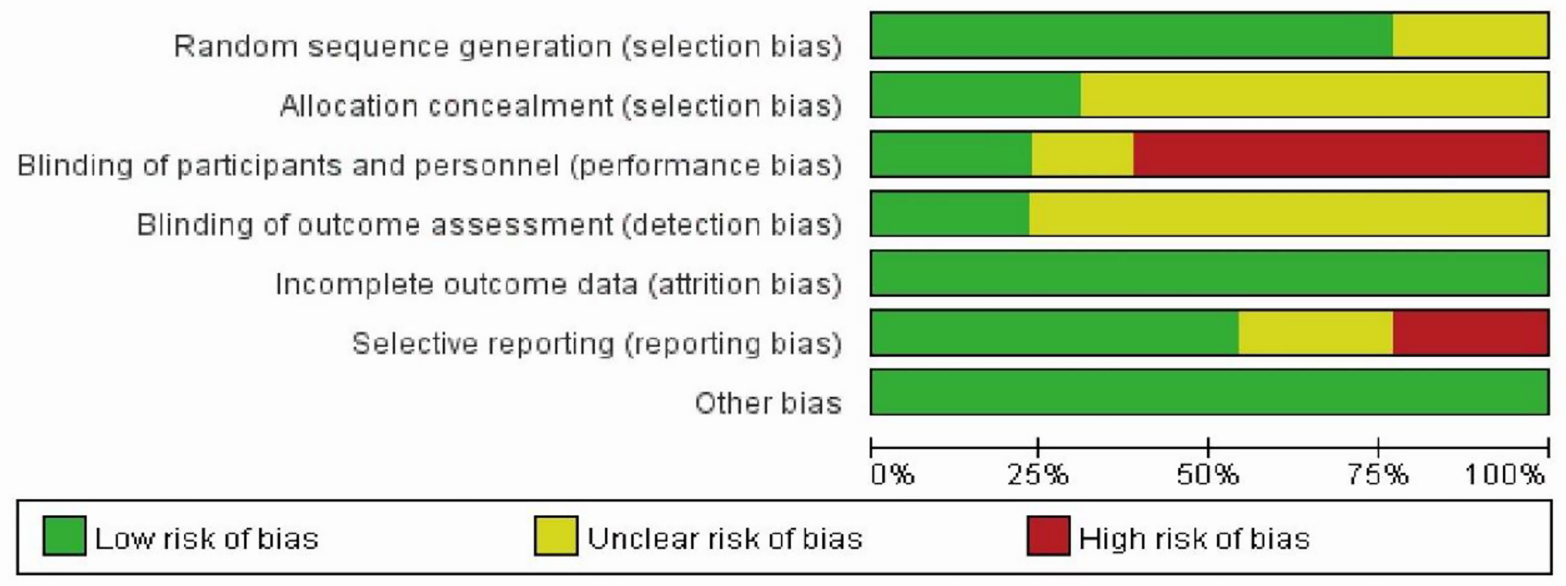
Risk of bias for included studies.

The risk of bias in 13 RCTs is shown in **Figure 2**. Ten trials (20–22, 27, 28, 30–34) had a low ROB regarding random sequence generation, whereas the remaining RCTs (20, 29, 30) did not report the methods. Four RCTs (21, 26, 28, 33) had a low risk of allocation concealment with opaque envelopes. The highest risk of bias in eight studies (21–23, 30–34) was due to the absent masking of participants and personnel. Only two trials (26, 27) demonstrated blinding for outcome assessment. Three RCTs (32–34) had high ROBs with selective reporting of outcomes for incomplete baseline characteristics of cancer survivors and results of trials.

### 3.3 Analysis of Overall Effects

#### 3.3.1 Short-Term Effect

**Figure 3:**
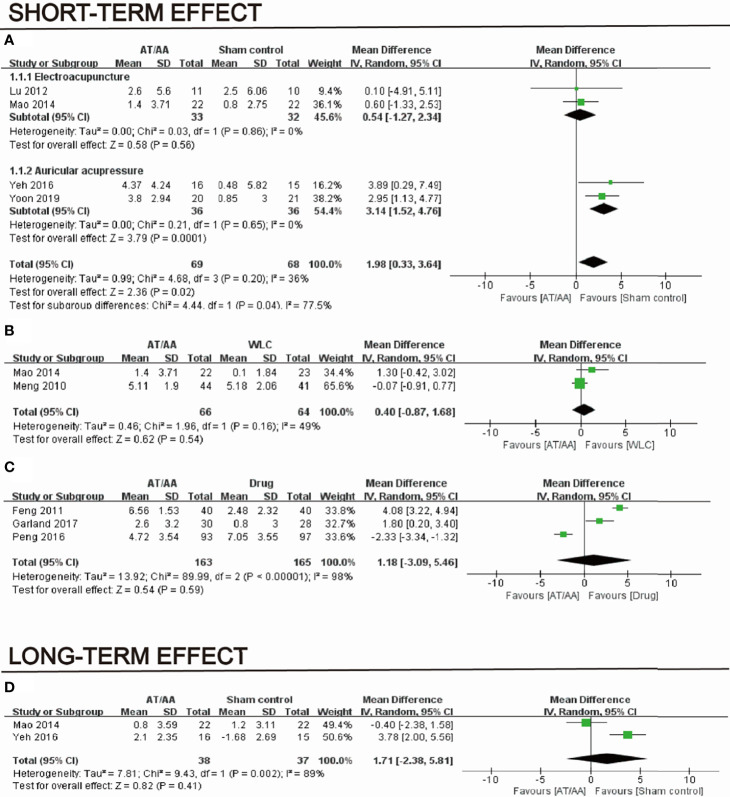
Short-term effect of acupuncture and auricular acupressure groups vs. **(A)** sham, **(B)** wait-list, and **(C)** drug and **(D)** long-term effect of acupuncture and auricular acupressure groups vs drug.

Four RCTs with a total of 137 patients provided adequate data on the insomnia scale scores and were included in the meta-analysis (20, 27–29). Unsurprisingly, it reported that sham controls was superior to acupuncture and AA groups in the improvement of sleep quality, with mean differences of 1.98 (95% CI = 0.33, 3.64; *p* = 0.02; *I^2^* = 36%; **Figure 3A**). Compared to the wait-list control, the pooled results of two RCTs (130 patients) suggested no significant reduction in acupuncture on the score of sleep scales (MD, 0.40; 95% CI=−0.87, 1.68; *p* = 0.54; *I^2^* = 49%; **Figure 3B**). Meta-analysis of three studies (328 patients) showed equivalent effects of acupuncture therapy as drug therapy on the score of sleep scales with substantial heterogeneity (MD, 1.18; 95% CI=−3.09, 5.46; *p* = 0.59; *I^2^* = 98%; **Figure 3C**).

#### 3.3.2 Long-Term Effect

Two sham-controlled RCTs showed no significance of acupuncture or AA on insomnia scale scores (MD, 1.71; 95% CI=−2.38, 5.81; *p* = 0.41; *I^2^* = 89%; **Figure 3D**).

#### 3.3.3.Subgroup Analyses

When compared to sham control, subgroup analyses suggested no evidence that AA (MD, 3.14; 95% CI=1.52, 4.76; *p* = 0.0001; *I^2^* = 0%) and acupuncture (MD, 0.54; 95% CI=-1.27, 2.34; *p* = 0.56; *I^2^* = 0%) were associated with the reduction in insomnia scale scores. The heterogeneity degraded from 36% to 0%.

#### 3.3.4 Sensitivity Analysis

**Figure 4:**
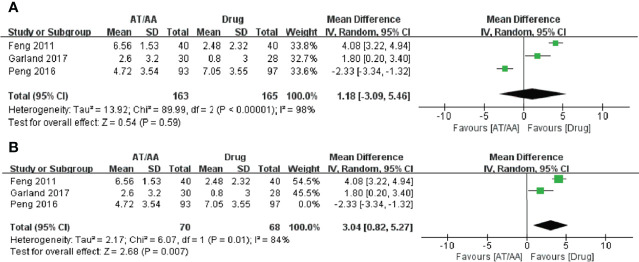
Forest plots of the **(A)** short-term effect and **(B)** long-term effect of acupuncture or auricular acupressure for cancer-related insomnia.

Sensitivity analysis was realized by subgroup analysis; drug-controlled RCTs demonstrated no significance for short-term effect but showed long-term effect on sleep scales in studies with unclear or low risk of selection bias (MD, 3.04; 95% CI=0.82, 5.27; *p* = 0.007; *I^2^* = 84%; **Figures 4A, B** **)**.

#### 3.3.5 Publication Bias

Funnel plots were not analyzed due to the small number of eligible studies.

#### 3.3.6 Safety of Acupuncture and Acupressure

Minor adverse events were reported in one study (21, 29) (**Table 3**), in which patients complained about minimal, adverse effects from the AA. All adverse events were mild to moderate. Seven RCTs reported no adverse effects (20, 22, 23, 26, 27, 30, 32), while adverse events were not mentioned in other studies (28, 31, 33, 34).

## 4 Discussion

This is the first review and meta-analysis to investigate the efficacy and safety of acupuncture and AA in sleep disturbances of cancer survivors. The results of the meta-analysis indicated that cancer survivors treated with acupuncture and AA might not experience a positive effect on sleep quality when compared to sham control, wait-list control, or drug therapy, which is inconsistent with the previous studies (16, 19). The difference might be owing to the following aspects: first, recent high-quality studies enrolled and strict inclusion criteria applied. Data of two trials (17, 18) included in the research (19) were from the same team, and they were replaced by a recent study (34) of their team in this research. Second is the generally high ROBs for methodological limitations of acupuncture and AA trials. Lastly is the high heterogeneity of using various acupoints, frequency, and duration.

Although 13 studies had a total sample of 961 participants and were conducted in six countries, which included United States, China, South Korea, Denmark, Sweden, and Australia, acupuncture and AA with few adverse effects were poor persuasion for cancer survivors (35). There was a study suggesting that lack of related knowledge was a major barrier in the use of acupuncture on common cancer symptoms (36); cancer survivors generally were confused about what symptoms or medical conditions that acupuncture could be used to treat, how acupuncture works, and how long the effects of acupuncture lasts (37). To address misconceptions above, we need to provide appropriate and timely education and communication in the treatment of insomnia for cancer survivors (37, 38). Certainly, robust evidence is the key factor based on evidence-informed patients, as participants preferred the treatment perceived as having stronger evidence (35). Thus, more high-quality clinical studies and detailed guideline of cancer survivors management should be promoted.

We found that the combination of acupuncture or AA with drug or sleep hygiene practices could significantly improve sleep quality of cancer surviors, and it was reported that participants responded to acupuncture effectively with responsible sleep hygiene practices and persistence of a durable therapy (39). Besides, there were studies showing that the combination of drug with sleep hygiene practices helped alleviate the patients’ depression (22, 23). Therefore, acupuncture or AA should be combined with sleep hygiene practices for the sleep management of cancer survivors, which might be more effective. Furthermore, the persistence of acupuncture contributes to a long-term effect (39, 40).

The negative results from sham-controlled RCTs suggested the potential efficacy of true and sham acupuncture or acupressure in improving sleep quality in cancer patients. The points of sham acupuncture employed in a number of included trials were not associated with improving sleep, so it was not physiologically inserted. In some sham control, needles were even retractable, but they could still elicit effects in participants. In reality, psychological effect was inevitable in the course of sham acupuncture for insomnia in cancer patients, while psychology was strongly linked with insomnia disorder (41). This might be another contributing factor in the review findings showing no significance between the two groups on improvement of sleep disturbance in cancer patients.

Subgroup analyses suggested the potential efficacy of AA in improving sleep quality for cancer survivors. The possible mechanism reported was that auricular reflective points were connected to the internal organs of the body (20, 42). Meanwhile, auricular therapy has been widely used to relieve symptoms like pain with few side effects (20, 43). However, we need more RCTs to illustrate the efficacy of AA on insomnia due to a paucity of related literature so far.

The drug-controlled RCTs had high heterogeneity in the study. The result of the sensitivity analysis suggested that acupuncture had superior short-term effects in studies with unclear or low risk of selection bias, and the statistical heterogeneity just fell from 99% to 84%. The fluoxetine- and gabapentin-controlled trials showed that they could improve insomnia by alleviating other symptoms causing insomnia in cancer survivors (31, 33), and it is well-known that fluoxetine and gabapentin were used for cancer-related depression and pain, respectively (44, 45). Besides, differences among drug comparators (i.e., manufacturers, dose, frequency, and course) were also factors leading to heterogeneity. Thus, studies on the specific drug-controlled group would be necessary to provide robust evidence of the acupuncture effects.

There were some other surprising findings in the study. A majority of the trials included were conducted in women, which might be closely related with characteristics of cancer survivors who are suffering from sleep disturbances (46, 47); for example, the prevelance of insomnia was lower in men with prostate cancer (25%–39%) but higher in women with breast (42%–69%) and gynecological (33%–68%) cancer (46). In addition, there was a study confirming that breast cancer survivors had higher prevelance of insomnia during chemotherapy (47). To the best of our knowledge, cancer treatments are also one of the predisposing causes of insomnia (48). Subgroup analysis about patients’ features was not conducted due to a limitation of included trials in each group. Further research could focus on the relationship of the treatments with specific cancer patients’ insomnia.

This review and meta-analysis is not without limitations. First, given the limited number of CBT-controlled RCTs in each group, it was not possible to compare the efficacy of acupuncture and AA when compared to comparators like CBT. Second, the study used subjective scales (e.g., PSQI) instead of polysomnogram as outcome measure due to few relevant literature, which reduced the credibility of this conclusion. Lastly, funnel plots evaluating publication bias was not feasible owing to the small number of included studies. In short, future RCTs are needed to provide robust evidence of the association of acupuncture or AA with the improvement of sleep disturbances of cancer survivors.

## 5 Conclusions

The findings of this systematic review and meta-analysis suggest that acupuncture or AA is irrelevant to the improvement of sleep disturbances in cancer survivors for short- or long-term effects. Adverse events were minor. To further understand the potential role of acupuncture and AA on cancer-related insomnia, well-designed and large-scale randomized controlled trials are needed.

## Data Availability Statement

The original contributions presented in the study are included in the article/supplementary material. Further inquiries can be directed to the corresponding author.

## Author Contributions

QW and SL handled conception, design of the review, data analysis, and drafting of the manuscript. XW, QT, HX, and SZ carried out data extraction and assessment of risk of bias. QF and HC were responsible for administrative support and supervision. RP and WW performed critical revision of the manuscript for important intellectual content and participated in conception and design. All authors contributed to the article and approved the submitted version.

## Conflict of Interest

The authors declare that the research was conducted in the absence of any commercial or financial relationships that could be construed as a potential conflict of interest.

## Publisher’s Note

All claims expressed in this article are solely those of the authors and do not necessarily represent those of their affiliated organizations, or those of the publisher, the editors and the reviewers. Any product that may be evaluated in this article, or claim that may be made by its manufacturer, is not guaranteed or endorsed by the publisher.

## Abbreviations

RCTs, randomized controlled trials; AT, acupuncture; EA, electroacupuncture; SA, sham acupuncture; AA, auricular acupressure; WLC, wait-list control; CBT, cognitive behavior therapy; PSQI, Pittsburgh Sleep Quality Index; WHQ, Women’s Health Questionnaire; QLQ-C30, cancer-quality of life questionnaire-core 30; MDASI, M.D. Anderson symptom inventory; QOL, quality of life. E/C, Experimental group/control group; ISI, insomnia severity index; NR,not reported; RevMan, Review Manager Software; CI, confidence interval; MD, mean difference.
